# Leveraging expectation effects to improve outcomes in the context of digital mental health interventions

**DOI:** 10.1016/j.invent.2026.100986

**Published:** 2026-08-08

**Authors:** Carmen Schaeuffele, Stephanie Haering, Christina Kampisiou, Yvonne Nestoriuc, Marcel Wilhelm, Winfried Rief, Christine Knaevelsrud

**Affiliations:** a Freie Universität Berlin, Dept. of Education and Psychology, Clinical-Psychological Intervention, Habelschwerdter Allee 45, 14195, Berlin, Germany; b Philipps University of Marburg, Clinical Psychology and Psychotherapy, Gutenbergstr. 18, 35032, Marburg, Germany; c Helmut-Schmidt-Universität/University of the Federal Armed Forces Hamburg, Clinical Psychology and Psychotherapy, Holstenhofweg 85, 22043, Hamburg, Germany; d German Center for Mental Health (DZPG), Partner site Berlin -Potsdam, Germany

**Keywords:** Digital mental health intervention, Expectations, Placebo effects, Nocebo effects, Observational learning, Therapeutic alliance, Personalization, Persuasive design

## Abstract

Patients' expectations about treatment benefits are robust predictors of clinical outcomes across medical and psychological contexts. Recent evidence suggests that expectancy effects in digital mental health interventions (DMHIs) are comparable in magnitude to those observed in face-to-face treatments. Despite their relevance, systematic guidance on how expectations can be shaped and optimized within DMHIs is limited. This narrative review summarizes theoretical and empirical work on expectations, including placebo and nocebo mechanisms, and digital intervention design to outline how expectation principles may be integrated into DMHIs. We describe three overarching principles - proactive expectation management, warmth and competence, and observational learning - and discuss how these mechanisms can be translated into practice across different stages and components of DMHI, including recruitment and onboarding, content, guidance, and interface design. We further highlight opportunities for expectation monitoring, automated feedback, and just-in-time adaptive interventions to support expectations. While modifications to single components may yield limited effects, coordinated, expectation-informed optimization across multiple DMHI components may have the potential to meaningfully enhance engagement and clinical outcomes. We conclude by discussing conceptual and methodological considerations and outline promising future directions for integrating the expectation lens within digital mental health.

## Highlights

• This narrative review introduces three key principles to optimize expectation effects in DMHIs.
• The three principles include expectation management, warmth and competence, and observational learning.
• The paper highlights how these principles can be applied across recruitment, onboarding, content, guidance, and design.
• It highlights opportunities for expectation monitoring and automated, personalized feedback in digital interventions.

## 1 Background

### 1.1 The importance of expectations for mental health outcomes

Patients enter treatment with beliefs about what treatment will bring about, commonly referred to as expectations. These expectations can concern the anticipated benefits of treatment (positive outcome expectations), absence of improvement or worsening of symptoms (negative outcome expectations), or the occurrence of adverse effects (side effect expectations). Positive outcome expectations have received the most empirical attention and have consistently been shown to be relevant for treatment outcomes across a wide range of conditions and interventions. In psychotherapy research specifically, patients' expectations of the treatment have long been recognized as a common factor influencing treatment outcome (Grencavage and Norcross, 1990). This association has been substantiated meta-analytically: In the most comprehensive synthesis to date, Constantino et al. (2018) found that more optimistic pretreatment or early-treatment outcome expectations were associated with more favorable psychotherapy outcomes across 81 independent samples (N = 12,722), with a statistically significant and small effect (r = 0.18 or d = 0.36).

Psychological interventions are increasingly delivered via the internet, and growing evidence indicates that digital mental health interventions (DMHIs) yield outcomes comparable to traditional face-to-face treatments across a variety of clinical conditions (Hedman-Lagerlöf et al., 2023). Regarding outcome expectations in DMHIs, a recent individual patient data meta-analysis found comparable expectancy effects for DMHI versus face-to-face psychotherapy (Pontén et al., 2023): They found outcome expectations predicted outcomes without a moderating effect of the DMHI or psychotherapy format (β = 0.27). Their findings suggest that outcome expectations in DMHIs may be as important as in face-to-face psychotherapy.

Beyond their association with treatment outcomes, expectations also matter earlier in the DMHI pathway. Acceptance of DMHIs is a key facilitator of their implementation in routine care but is often low (Philippi et al., 2021). Drawing on the Unified Theory of Acceptance and Use of Technology (UTAUT), Philippi et al. (2021) validated an acceptance model across ten studies (N = 1588) and found that performance expectancy, the expected benefit of using the DMHI, was the strongest predictor of acceptance (γ = 0.68, p < .001), outweighing effort expectancy, social influence, and facilitating conditions. Because acceptance is a necessary precondition for any DMHI to exert an effect, this suggests that expectations may be a leverage point not only for improving outcomes among patients already in treatment, but also for increasing the reach of DMHIs more broadly.

Unlike many established predictors of treatment outcome, such as symptom severity or chronicity, expectations are considered a malleable, state-like construct that can plausibly be shaped by interventions, making them a particularly actionable target for improving both engagement and outcomes in DMHIs. Integrating an expectation-focused perspective into DMHI development and implementation may therefore provide promising targets to further improve clinical outcomes and optimize healthcare efficiency. To date, however, the knowledge about the expectation-outcome link has rarely been translated into systematic strategies for fostering adaptive expectations within DMHIs. Therefore, the goal of the present review was to summarize strategies from the extant empirical literature to address and optimize patients' expectations within DMHIs with the ultimate goal to improve outcomes and engagement.

### 1.2 General expectation-optimizing principles: how can expectations be improved?

A range of expectation-influencing strategies have been proposed in the psychotherapy literature (Constantino et al., 2012) and extended to the psychotherapeutic context by placebo/nocebo research (Rief and Wilhelm, 2024). In this narrative review, we focus on three principles that operate at complementary levels and are each particularly well-suited to the digital context: (1) proactively managing positive, negative, and side effect expectations, (2) warmth and competence and (3) observational learning. We focus on these three principles because they are particularly relevant and scalable within digital contexts: proactive expectation management serves as a meta-principle applicable across all stages of an intervention, from recruitment and onboarding through automated monitoring; warmth and competence addresses the relational deficit inherent to digital interventions; and observational learning is readily operationalized through digital content in ways that are difficult to scale in individual therapy. In a first step, we will review existing evidence on these principles from the broader psychotherapy, placebo/nocebo and DMHI literature before deriving specific recommendations for applying these principles across different DMHI components.

#### 1.2.1 Proactive expectation management

Patients' expectations about a treatment outcome are multifaceted. Expectation effects linked to beneficial outcomes are known as placebo effects, whereas nocebo effects describe harmful expectation-related outcomes (Colloca and Barsky, 2020). Accordingly, outcome expectations may involve positive (e.g., “This treatment will help improve my condition”), negative (e.g., “My condition might get worse”) and side effect expectations (e.g., “I'm worried about experiencing side effects”) (Colloca and Barsky, 2020). Research on placebo effects in the biomedical field has shown the powerful role of positive expectations, explaining up to 80% of the treatment effect in the active drug group (Meyerson et al., 2023). Within psychotherapy research, positive outcome expectations have also been linked to improved treatment outcomes (Wilhelm et al., 2025). For example, in a randomized controlled trial of psychological interventions for pain, the proportion of patients who experienced positive treatment outcomes was more than twice as high for those who initially reported positive outcome expectations (Beasley et al., 2017). Accordingly, fostering positive outcome expectations is central to harnessing treatment effectiveness.

Negative outcome expectations must not be neglected, however. Within psychological pain interventions, patients who expected no change or symptom worsening more commonly experienced negative treatment outcomes (Beasley et al., 2017). Research suggests that prior (unsuccessful) treatment experiences might be particularly relevant in forming negative outcome expectations (Zunhammer et al., 2017). Exploring prior treatment experiences and differentiating them from the new treatment approach hence can help to improve existing negative beliefs.

Side effects within psychotherapy typically include the perception of one's own problems as more complex than before, symptom worsening, experiencing new symptoms or interpersonal conflicts (Wittmann et al., 2025). Findings from nocebo research suggest that the framing of nocebo effects may alter patient's side effect expectations. For instance, instead of communicating the proportion of patients who experience a specific side effect (negative framing, e.g. “5% of participants report side effects following this treatment”), communicating the proportion of patients who do *not* experience this side effect (positive framing, e.g. “95% of participants do not report side effects following this treatment”) has been linked to reduced side effect expectations (Bender et al., 2023). Another promising approach includes framing side effects as “onset sensations”, that indicate that the treatment is starting to work (Fernandez et al., 2019) – or, in the case of psychotherapy – normalize them as sign that the patient is addressing and dealing with difficult thoughts, behaviors or emotions (e.g. “it is common that patients experience an increase in symptoms at the beginning of therapy. This shows that they are actively dealing with difficult topics, thoughts and behaviors”). Interestingly, positive, negative and side-effect expectations exist independently of each other (Basedow et al., 2023). Accordingly, proactively fostering positive, and mitigating negative and side effect expectations is crucial to enhance intervention effectiveness. Informing patients about possible side effects of psychotherapy using such framing strategies has been shown to increase positive expectations and therapy motivation while decreasing decisional conflicts before the start of psychotherapy (Gerke et al., 2024).

#### 1.2.2 Warmth and competence

Research on placebo effects furthermore highlights that besides directly addressing expectations, the remaining common factors of a therapist-patient interaction should not be ignored. For example, a video experiment on changing negative expectations and fostering positive expectations found that this is best achieved by a therapist that is both warm and competent (as opposed to being perceived as warm only or competent only) (Seewald and Rief, 2023a). Ultimately, this research highlights the role of the therapeutic alliance in – indirectly - augmenting expectation effects.

The therapeutic alliance – including (i) the formation of a *bond* between patient and provider, (ii) mutual agreement on the treatment *goals*, and (iii) the treatment process and steps towards achieving the goals, i.e. *tasks* (Bordin, 1979) – is also of relevance in DMHI: Users establish a bond and agreement with tasks and goals to a similar extent to conventional psychotherapy (Berger, 2017, Berger, 2025). Importantly, while the bond in guided interventions may refer to the bond to the mental health care provider guiding the users, the other alliance components, i.e., agreement with task or goals, refer to the parasocial relationship with the digital component (Berger, 2017). Similarly to conventional psychotherapy, alliance in DMHI is linked to outcomes (Kaiser et al., 2021; Van Lotringen et al., 2021).

As in conventional provider-patient interactions, the DMHI may transport warmth and strengthen the bond by using empathic, understanding, and personable language (Laferton et al., 2025). The bond of a user with the DMHI may also be increased by the fit of the user's preference and complaints with the DMHI's therapeutic content (Berger et al., 2014). Accordingly, personalization of content may increase a user's bond to the DMHI and potentially also their expectation that the DMHI will benefit them (Barazzone et al., 2012).

Competence in DMHIs may be conveyed through the same pathways as in other patient-provider interactions: transparent presentation of scientific evidence, such as referencing the intervention's evidence base, highlighting alignment with current treatment guidelines, or citing relevant studies when delivering recommendations may be beneficial (Laferton et al., 2025). Competence may furthermore be fostered through well-structured communication and affirmation of expertise in the suggested treatment, such as references to successful interventions in the past. As outlined above, however, demonstration of competence must go hand in hand with conveying professional warmth. Both clinical observational as well as experimental studies indicate that the best treatment outcomes are observed if providers are perceived as both warm and competent – as opposed to warm only or competent only (Kaptchuk et al., 2008; Seewald and Rief, 2023b, Seewald and Rief, 2023a).

#### 1.2.3 Observational learning

A third principle to target patient expectations is through observational learning (OL). OL refers to acquiring or modifying behaviors or beliefs through observing others (Bandura, 1977) and this technique can also be applied in the context of treatments. Observing others benefitting from treatment may influence placebo and nocebo effects across mental and medical conditions (Klauß et al., 2024). OL can be delivered through text-, video- and face-to-face interventions, with video- and face-to-face induced OL resulting in comparable effects superior to text-based OL (Klauß et al., 2024). Many DMHI apply OL implicitly by including testimonials, client stories, narratives, personas or case examples. In persuasive system design theory, the principle of social learning emphasizes the strategy of observing other users' engagement with the DMHI with the goal to increase usage and adherence (Kelders et al., 2012; Mutter et al., 2024; Oinas-Kukkonen and Harjumaa, 2009). This may be extended to foster positive and mitigate negative outcome expectations as well. For instance, video-based patient stories that portray realistic experiences and treatment benefits may serve as powerful models for shaping users' expectations throughout the treatment (for an example from the medical context in total knee replacement see Stuhlreyer et al., 2022 and from changing attitudes towards psychotherapy see Braun-Koch et al., 2022). Drawing on broader research on expectancy effects, it is important that such representations foster realistic rather than overly optimistic expectations, as unrealistic expectations can undermine effects (Rief, 2022). In digital settings, where therapist contact is limited, OL can play a crucial role in maintaining this “corridor” of realistic expectations. Presenting authentic patient narratives may help users form a nuanced understanding of improvement, illustrating that recovery often involves ongoing challenges alongside overall progress. Moreover, such stories can normalize temporary setbacks and model adaptive coping. At the same time, case examples should be carefully designed in order to prevent unwanted induction of nocebo effects (Meeuwis et al., 2023).

## 2 Applying expectation principles to DMHI components

The aforementioned principles can be applied across a variety of DMHI components. The following section gives an overview of how considerations within (a) the recruitment and onboarding process of a DMHI, (b) its content, (c) guidance, and (d) design may help to translate the expectation optimizing principles into DMHI practice. An overview of general recommendations as well as application examples for the DMHI context is presented in Table 1. It should be noted that while the three overarching principles are grounded in the empirical literature reviewed above, the specific translations into DMHI components presented in Table 1 have, for the most part, not themselves been empirically evaluated. These recommendations represent theoretically and empirically informed suggestions for how the underlying principles might be operationalized in practice.

**Table 1:** Recommendations on how to leverage the expectation principles across DMHI components.

Expectation principle	General recommendation	Recruitment and onboarding	Content	Guidance	Design aspects
Proactive Expectation Management	• Explore patients' expectations • Foster positive outcome expectations • try to understand the development of negative and side effect expectations; show empathy for them with the aim to reduce them • if a patients report negative prior experiences with the treatment, validate those, highlight differences between the new and old treatment and refer back to them throughout	• Explain the treatment rationale • Provide non-technical information about the evidence base for the treatment • Proactively address potential DMHI-specific concerns • Consider a positive and normalizing framing of potential side effects • Consider standardized assessment of patients' expectations to provide tailored onboarding options	• Include psychoeducation about treatment efficacy and the expectation-outcome link • Consider integrating expectation-focused psychological interventions	• Proactively address dynamic changes in expectations • Instill hope • Reference the user's experience with benefits, worsening and negative effects and give personalized and normalizing feedback	• Proacitvely use charts to visualize efficacy information • Use flow diagrams to visualize and enforce psychoeducation about the expectation-outcome link
Warmth & Competence	• Use empathic, understanding and personable language • Present scientific evidence for the treatment • Highlight professional expertise and qualification • Structure communication clearly	• Avoid recruitment efforts or claims that might be perceived as manipulative or insincere • Display of professional credentials and affiliations (where applicable) can build initial trust	• Convey warmth through personable and relatable descriptions • Convey competence by science-backed, experience-informed explanations	• Use language that is warm and understanding • Use “alliance-bolstering” phrases • Consider integrating self-disclosure where appropriate • Convey competence by referencing scientific evidence • Convey competence by introducing your professional credentials	• Tailor look and feel of the intervention to your target user group • Consider using warm and gamified features • Consider adding institutional logos to convey competence
Observational Learning (OL)	• Implement observational learning elements purposefully to shape positive and realistic expectations, support adherence, and model adaptive coping. • Evidence suggests that video-based observational learning is more effective than text-based observational learning therefore, prefer short, credible video stories that depict treatment benefits and progress rather than only app use or engagement behavior. • Observational learning should focus on realistic improvement trajectories and be framed carefully to avoid unintended nocebo effects.	• Introduce observational learning early through video-based onboarding materials showing realistic patient journeys and benefits. • Present authentic testimonials that normalize effort and progress, not just success stories. • Consider including specific case examples in the onboarding procedure. This may strengthen placebo-related mechanisms more effectively than abstract statistics. • Consider the power of users' (positive and negative) social media engagement and monitor and manage online comments and discussions related to the DMHI	• Include preferably video-based patient stories that portray realistic experiences and treatment benefits. • Present narratives not only at the beginning but also throughout the intervention. • Use stories to illustrate ongoing challenges alongside improvement, helping users understand what “getting better” can look like. • Narratives can normalize setbacks and model adaptive coping • Refer to the effect of the testimonials (“at first I was skeptical if this app can help me. But when I read about how others perceived it and engaged with it, I thought I should give it a try”) • Careful framing is necessary to avoid modeling maladaptive experiences that could amplify disappointment and nocebo effects.	• Embed symbolic vicarious learning by describing other patients' successful experiences • Include brief, credible case examples to foster a sense of attainability and reinforce adherence	• Support observational learning through specific UX elements such as characters or scenarios that allow users to learn by example (e.g., a gamified intervention where users help a character overcome problems). • Consider user-centered development to ensure observational learning is appropriate and helpful for the target population

*Note*. Recommendations are derived from and grounded in the theoretical and empirical literature discussed in the main text on placebo/nocebo mechanisms and the **e**xpectation-optimizing principles. However, most specific applications listed here have not been directly empirically tested within DMHI contexts and should be considered translational suggestions rather than tested evidence-based guidelines. DMHI Digital mental health intervention, OL observational leaning, UX user experience.

### 2.1 DMHI component: recruitment and onboarding

Similarly to face-to-face treatments, early communication about the DMHI treatment should instill hope and positive expectations among potential patients. Recruitment and onboarding may shape these early expectations and should be handled deliberately. Explaining the (digital) treatment rationale will allow patients to understand the intended mechanisms of action and thus help to expect that the DMHI will work for them (Norcross and Lambert, 2018). Furthermore, such explanations may address potential concerns that relate to negative outcome expectations. For example, a typical concern around DMHIs involves data safety issues (Taher et al., 2025). Explaining the data privacy safeguards and highlighting the possibility of anonymous DMHI use (where applicable) can address potential reservations about the treatment. Displaying professional credentials and affiliations can further strengthen perceptions of competence and authority and build initial trust (Sbaffi and Rowley, 2017).

Positive expectations might furthermore be fostered through an accessible, non-technical summary of the evidence base of the treatment, e.g. stating that the treatment approach is supported by much research and that people using the intervention tend to get significantly better. In addition to such general reviews of the treatment findings, providers might draw upon more personal OL processes and provide realistic video-based examples of patients using the intervention and benefitting from it. Using such expectation-optimized OL interventions can improve outcome expectations even among treatment-critically minded individuals (Braun-Koch and Rief, 2022).

In this regard, newer research further emphasizes the relevance of online commenters' reactions to the claims presented in a DMHI advertisement (Kareklas et al., 2015). Accordingly, it is crucial that DMHI providers' persuasive efforts are not perceived as manipulative or insincere, resulting in negative community feedback. On the other hand, positive community feedback (e.g. prior users commenting favorably on the DMHI) are a real-live example of OL that may benefit others' positive outcome expectations. Such community-based expectation effects will likely become more and more important given the growing use of online recruitment through social media platforms such as Instagram or TikTok (Oudat and Bakas, 2023). Accordingly, Kareklas et al. (2015) encourage health care providers to carefully monitor and manage online comments to their products and to be open for discussing relevant topics, e.g., answering questions about the DMHI or replying to potential concerns that are expressed.

In addition to the aspects outlined above, which might be relevant during both recruitment and onboarding, the onboarding phase offers further valuable targets to engage with and address a user's individual expectations. Gerke et al. (2024), for instance, developed an optimized informed consent (OIC) procedure that addresses many of the above described techniques (e.g. explanation of the treatment rationale, provision of the evidence base, framing and visualization of efficacy and side effects information) and proactively inquires and address an interested participant's positive, negative, and side effect expectations as well as prior treatment experiences. While Gerke et al. (2024) explore these topics within a personal conversation, DMHIs offer exciting possibilities to systematically assess patients' differential expectations and experiences through validated instruments and provide standardized expectation-enhancing interventions tailored to the person's needs. While such a personalized onboarding procedure has not yet been tested in DMHIs, it would allow to disentangle actual expectation effects from other factors related to face-to-face interventions (Gruszka et al., 2019).

### 2.2 DMHI component: content

The DMHI's therapeutic content is most typically organized in different treatment modules and conveyed in the form of psychoeducation, interactive exercises, and reflection prompts. Expectations evolve over time: A longitudinal study found that only expectations later in treatment, not at baseline, were associated with depression outcomes (Wälchli et al., 2025). Similarly, later expectations appear to be more strongly related to outcomes than earlier expectations (Pontén et al., 2023). Thus, baseline expectations may reflect general attitudes towards a DMHI, whereas later expectations are shaped by users' direct experiences with the current DMHI (Wälchli et al., 2025). Given that expectations fluctuate through DMHIs, it is crucial to monitor expectations and actively support them across modules.

There are several ways in which a DMHI's content can target the expectation-related principles. First, the DMHI can explicitly introduce psychoeducation about expectations and their relationship to outcome, enabling users to recognize and intentionally work with their own outcome expectations (Michnevich et al., 2022; Nestoriuc et al., 2021). Second, personalization of content - aligning examples, exercises, and explanations with users' concerns or preferences – may enhance the perceived fit with the DMHI (Schaeuffele et al., 2025b). Personalization of content may increase a user's alliance to the DMHI and in turn increase their expectation that the DMHI will benefit them. Third, all written content can be expectation-optimized, for example by conveying warmth through personable descriptions and conveying competence by science-backed, experience-informed explanations. Fourth, the therapeutic content itself can explicitly target disorder-specific dysfunctional expectations and incorporate expectation-violation procedures, as demonstrated in expectation-focused psychological interventions (Ewen et al., 2023; Wilhelm et al., 2022). Such an approach helps users test and revise maladaptive beliefs and may further strengthen adaptive outcome expectations.

### 2.3 DMHI component: guidance

DMHI can be guided or unguided. Guidance is implemented in DMHI to enhance adherence and most commonly consists of written asynchronous feedback provided by a trained mental health professionals (Krieger et al., 2023). A recent scoping review investigated therapist behaviors in guided IMI (González-Robles et al., 2024). While only one study reported a therapist behavior (“instilling hope”) directly targeting expectation effects, several of the identified therapist behaviors may indirectly support expectations.

Guidance can influence expectations by directly addressing user's beliefs about treatment and outcome. Therapists may use expectation-relevant information, collected through writing tasks or validated expectation-focused questionnaires (e.g. with the 1-item GEEE) (Rief et al., 2021), to address any concerns or misconceptions that might exist and strengthen helpful beliefs about treatment outcome. To achieve this, therapists may strategically embed OL to strengthen placebo-related mechanisms. Even without direct observation, “symbolic vicarious learning” can occur when therapists describe other patients who faced similar challenges and benefited from engaging with the DMHI (Bandura, 1977). Providing brief, credible accounts of others' improvements, may help users to view progress attainable and strengthen their engagement. Guidance may also influence expectations by shaping the relational context of the intervention. Therapists' messages can convey empathy and understanding, strengthening the perceived bond between user and therapist. Guidance can also signal competence, for instance, when therapists introduce their qualifications or support recommendations with clinical experience or scientific evidence. Communicating warmth and competence in this way can enhance trust in both the therapist and the DMHI, indirectly supporting positive expectations and engagement without explicitly addressing expectations.

Human therapists may not be necessary for implementing these principles, and expectancy-enhancing strategies - proactive expectation management, conveying warmth and competence and OL - can be delivered through automatic feedback in unguided DMHI to leverage scalability. Gruszka et al. (2019) for instance suggest that patient data on symptom improvement might be used for tailored expectation-focused information. For example, patients who show an early treatment response might receive positively reinforcing feedback (e.g. “You have been using the DMHI for a week now, and your symptoms have already decreased. We expect that symptoms will continue to decrease with regular use of the app.”). For users who do not show early improvement, expectations may be supported by acknowledging common experiences and offering a realistic trajectory (“Some patients at first did not seem to benefit from the DMHI, and a few even felt frustrated. Often, however, their symptoms improve bit by bit over time.”). This automated feedback can also convey warmth and competence and may even incorporate brief case examples to support observational learning. Although DMHIs provide exciting opportunities for systematically assessing and individually addressing outcome expectations, no study has yet implemented and tested such an automated approach towards expectancy interventions.

### 2.4 DMHI component: interface design

In DMHI, expectation-related mechanisms can also be supported through visual and interface design aspects. Elements such as logos, displayed credentials, color schemes, illustrations, and the overall style and interactivity can be selected with placebo mechanisms in mind. A DMHI's interface can be designed to communicate warmth (e.g., approachability, friendliness) and competence (e.g., professionalism, trustworthiness). The majority of such studies linking visual interventions features to the perceived warmth (approachability) and competence (trustworthiness) comes from general user experience (UX) or health-communication research rather than DMHI-specific studies. In a quasi-experimental study of online health-intervention stimuli, perceptions of simplicity (clarity and structure) and craftsmanship (topicality, sophistication and the professionalism of the design) significantly influenced perceived usefulness, ease of use, enjoyment and trust and these in turn influenced behavioral intentions to use the intervention (Denison-Day et al., 2023). Specific UX elements can also support OL by making learning from example more salient. For instance, in a study on a gamified eating disorder prevention, users help a digital character overcome their ED issues and by that learn by example (Karekla et al., 2022). Given the linear and “textbook-like” character of many DMHI, substantial opportunities remain to design UX features that more actively support OL. Finally, involving potential users in the design process through user-centered design can ensure that placebo-informed design choices, aimed at supporting warmth, competence, and OL, resonate with the target population.

## 3 Discussion and future directions

In DMHI research, isolating the effects of single components typically yields only small, incremental effects (Collins, 2018). For example, a recent factorial optimization trial of internet-delivered CBT for depression found that most individual components contributed only modestly to outcomes, with a small subset of ‘active ingredients’ accounting for the majority of the benefit (Watkins et al., 2023), suggesting that improvements to a single DMHI component alone are unlikely to produce substantial change. However, a DMHI that is systematically optimized across multiple levels and intentionally designed to shape, monitor, and address user expectations may have a more meaningful impact. Because expectations are consistently linked to treatment outcomes - higher expectations generally predict better engagement and symptom change - there is considerable potential in leveraging this knowledge to strengthen both outcomes of and engagement with DMHIs.

Importantly, while it is generally advised to foster positive expectations, DMHI providers should take caution not to induce unrealistically high outcome expectations. Newer research has demonstrated that the degree of expectation violations – i.e. the discrepancy between expected and actual outcome – may be related to symptom reduction in a non-linear way (Rief et al., 2022): If the expectation violation becomes too high, patients may be less likely to engage in an expectation change. Rather, exaggerated expectation inductions are at risk of cognitive immunization, which means that patients will ignore or deny the information e.g., by questioning the source credibility (“I cannot trust them”) or subtyping it (“This was just an exception”) (Rief et al., 2022). Accordingly, DMHI providers should induce positive, but still highly believable expectancy-effects among their users for example by aiming to balance outcome expectations with realistic side effects and coping expectations (Rief et al., 2023). We are currently investigating in an ongoing RCT whether expectation-optimizing telephone calls with the goal to improve low, bolster realistic, and potentially challenging overly optimistic expectations, may be beneficial for outcomes and expectations beyond supportive telephone calls (Schaeuffele et al., 2025a).

While outcome expectations are a significant predictor for successful treatment of both physical and mental conditions, mental disorders are further characterized by disorder-specific expectations such as negative expectations about the self (“I will not accomplish this”) or others (“They will not like me”) in patients with depressive disorders of catastrophizing thoughts (“I will get a heart attack”) in patients with anxiety disorders (Rief and Glombiewski, 2016). Expectation-focused psychological interventions (EFPIs) provide a structured approach to systematically integrate expectation-related knowledge into existing psychotherapeutic interventions to address disorder-specific dysfunctional expectations (Ewen et al., 2023; Rief and Glombiewski, 2016). For example, a recent randomized controlled trial examines the efficacy of expectation-focused cognitive behavioral therapy for depressed patients (Ewen et al., 2023). Within this approach, patients acquire knowledge on the link between expectations and depressive symptoms, relate their individual expectations to biographic experiences and perform behavioral experiments to test and actively influence dysfunctional disorder-specific expectations (Ewen et al., 2023). While it is beyond the aim and scope of this article, to explain EFPIs in detail, DMHI providers should be aware that re-evaluating their existing psychological interventions within an expectation-focused perspective might help to increase treatment effectiveness and better understand treatment failure (Rief and Glombiewski, 2016).

Using expectations as the overarching framework to guide improvements in DMHI design is helpful, but it is important to recognize that strategies intended to enhance expectations will probably rarely operate on expectations alone. Many expectation-focused features will likely have spillover or carry-over effects on related mechanisms, for example, strengthening the user's sense of connection or bond with the DMHI, increasing perceived credibility, or enhancing overall satisfaction (Barazzone et al., 2012). Moreover, (especially early) expectations may overlap with constructs like acceptance and intended usage (see literature on acceptance-facilitating interventions, e.g., Baumeister et al., 2015). These spillover and carry-over effects are not problematic when the primary aim is to enhance DMHIs in a broad, applied manner, where improvements across multiple therapeutic processes, outcomes and engagement with the DMHI are desirable. These issues only become limiting in fine-grained mechanistic research, where isolating the unique contribution of expectations may be the goal.

As discussed, integrating expectation-related principles into recruitment, onboarding, content and design of a DMHI, can be a powerful tool to make DMHIs even more effective. While this manuscript provides a first overview of possible ways of action, both the scientific understanding of how to optimize expectations in DMHIs and the translation of this knowledge into practice remain limited, and many promising possibilities have yet to be explored. Emerging technologies within DMHI, such as just-in-time adaptive interventions (JITAIs), may offer particularly promising contexts in which to test expectation-related principles empirically, given their capacity for standardized, continuous monitoring of patients' symptoms and expectations. Existing measures of expectations like the GEEE have been adapted to be applied more frequently as part of ecological momentary assessments (Nestoriuc et al., 2026). Utilizing these frequent assessments, JITAIs can be used explicitly to optimize expectations in a personalized and dynamic fashion, targeting critical moments as they arise in real time: For example, in case of symptom worsening after treatment initiation, providers might normalize the possibility of increased symptoms at the beginning of treatment and frame them as an indication that the user actively engages with and confronts personally difficult topics or providing a matching case example. In line with these opportunities, a recent study employs continuous expectation monitoring and JITAIs to explore and modulate nocebo effects in antidepressant discontinuation (Nestoriuc, 2025).

With the rise of mental health chatbots and accumulating evidence for their efficacy (Heinz et al., 2025; Hua et al., 2025) as well as their potential for sustained engagement in comparison to legacy DMHI, the question of their mechanisms of action arises. Thus far, mostly relational factors and a continuity of care have been emphasized (Siddals et al., 2024). Chatbots may also be particularly well suited to explore and challenge a patient's expectation through personalized and real-world conversations. Future research may investigate the role of expectations behind chatbots' effects as well as explore their potential to optimize expectations through real-time dialogue.

## 4 Conclusion

Systematically applying expectation-informed principles throughout a DMHI has the potential to improve user engagement and treatment outcomes. While the three principles reviewed here, proactive expectation management, warmth and competence, and observational learning, are grounded in theoretical and empirical work, their specific translation into DMHI components remains to be empirically investigated. Future work should prioritize systematically testing whether and how these principles and their proposed applications affect engagement and outcomes in DMHIs. Building on this evidence base, subsequent work can then move towards broader translation into scalable digital tools across diverse interventions and populations. Importantly, not only can expectation research benefit the DMHI field, but also vice versa. Because DMHIs allow expectation-related content and delivery to be standardized and manipulated with high precision, independent of provider-specific factors that are difficult to isolate in face-to-face treatment, they offer an ideal experimental platform for expectation research itself. DMHIs may thus help disentangle the unique contribution of expectation effects from provider or intervention effects more broadly in psychological treatments.

## Declaration of competing interest

The authors declare the following financial interests/personal relationships which may be considered as potential competing interests:

Christine Knaevelsrud, Yvonne Nestoriuc, Winfried Rief reports financial support was provided by German Research Foundation. If there are other authors, they declare that they have no known competing financial interests or personal relationships that could have appeared to influence the work reported in this paper.
